# Self-adjuvanted mRNA vaccination in advanced prostate cancer patients: a first-in-man phase I/IIa study

**DOI:** 10.1186/s40425-015-0068-y

**Published:** 2015-06-16

**Authors:** Hubert Kübler, Birgit Scheel, Ulrike Gnad-Vogt, Kurt Miller, Wolfgang Schultze-Seemann, Frank vom Dorp, Giorgio Parmiani, Christian Hampel, Steffen Wedel, Lutz Trojan, Dieter Jocham, Tobias Maurer, Gerd Rippin, Mariola Fotin-Mleczek, Florian von der Mülbe, Jochen Probst, Ingmar Hoerr, Karl-Josef Kallen, Thomas Lander, Arnulf Stenzl

**Affiliations:** Klinikum rechts der Isar der Technischen Universität München, Munich, Germany; CureVac GmbH, Paul-Ehrlich-Str. 15, Tuebingen, 72076 Germany; Charité University Hospital Berlin, Berlin, Germany; University Hospital Freiburg, Freiburg, Germany; Universitäty Hospital Essen, Essen, Germany; San Raffaele Scientific Institute, Milan, Italy; University Hospital of the Johannes-Gutenberg-University Mainz, Mainz, Germany; Ortenau Klinikum Offenburg-Gengenbach, Offenburg, Germany; University Hospital Göttingen, Göttingen/University Hospital Mannheim, Mannheim, Germany; University Hospital Schleswig-Holstein Campus Luebeck, Luebeck, Germany; Rippin-Consulting, Solingen, Germany; University Hospital Tuebingen, Tuebingen, Germany

## Abstract

**Background:**

CV9103 is a prostate-cancer vaccine containing self-adjuvanted mRNA (RNActive®) encoding the antigens PSA, PSCA, PSMA, and STEAP1. This phase I/IIa study evaluated safety and immunogenicity of CV9103 in patients with advanced castration-resistant prostate-cancer.

**Methods:**

44 Patients received up to 5 intra-dermal vaccinations. Three dose levels of total mRNA were tested in Phase I in cohorts of 3–6 patients to determine a recommended dose. In phase II, 32 additional patients were treated at the recommended dose. The primary endpoint was safety and tolerability, the secondary endpoint was induction of antigen specific immune responses monitored at baseline and at weeks 5, 9 and 17.

**Results:**

The most frequent adverse events were grade 1/2 injection site erythema, injection site reactions, fatigue, pyrexia, chills and influenza-like illness. Possibly treatment related urinary retention occurred in 3 patients. The recommended dose was 1280 μg. A total of 26/33 evaluable patients treated at 1280 μg developed an immune response, directed against multiple antigens in 15 out of 33 patients. One patient showed a confirmed PSA response. In the subgroup of 36 metastatic patients, the Kaplan-Meier estimate of median overall survival was 31.4 months [95 % CI: 21.2; n.a].

**Conclusions:**

The self-adjuvanted RNActive® vaccine CV9103 was well tolerated and immunogenic.

The technology is a versatile, fast and cost-effective platform allowing for creation of vaccines. The follow-up vaccine CV9104 including the additional antigens prostatic acid phosphatase (PAP) and Muc1 is currently being tested in a randomized phase IIb trial to assess the clinical benefit induced by this new vaccination approach.

**Trial registration:**

EU Clinical Trials Register: EudraCT number 2008-003967-37, registered 27 Jan 2009.

**Electronic supplementary material:**

The online version of this article (doi:10.1186/s40425-015-0068-y) contains supplementary material, which is available to authorized users.

## Background

Prostate cancer (PCa) remains the second leading cause of cancer death in men, accounting for 29,720 estimated cancer deaths in the US in 2013 [1]. While early diagnosis of PCa is associated with a 5-year disease-specific survival rate (SR) of 100 %, only 27.8 % 5-year SR is reported for patients with metastatic disease (SEER Stat Fact Sheet on prostate cancer, http://seer.cancer.gov). Advanced PCa is usually treated with hormone ablation therapy leading to tumor shrinkage [2]. However, tumors may relapse after a period of time ranging from a few months to several years at which time they progress into castration-resistant prostate cancer (CRPC). Treatment options for patients with metastatic CRPC include second generation anti-hormonal agents such as abiraterone or enzalutamide or palliative chemotherapy with docetaxel or cabazitaxel, which increase survival by 2–4 months [3]. In the past years, immunotherapeutic approaches have become more and more relevant. The cell-based therapeutic vaccine Sipuleucel T targeting the antigen PAP has been approved by the US Food and Drug Administration in 2010 and recently by the European Medicines Agency for the treatment of asymptomatic – minimally symptomatic metastatic CRPC based on a median prolongation in overall survival by 4.1 months compared to placebo controls [4]. Another prostate cancer vaccine against PSA, Prostvac-VF, has shown an improvement in median overall survival by 8.5 months in a double blind placebo controlled phase II trial [5].

Vaccination with messenger RNA (mRNA) encoding full-length tumor antigens is a novel option for immunotherapy. Early experiments showed that intradermal administration of mRNA led to protein expression and induction of humoral and cellular antigen-specific immune responses in mice [6–9]. In a phase I/II trial in patients with metastatic melanoma, direct intra-dermal injection of mRNA coding for relevant tumor-associated antigens was well tolerated and influenced the frequency of vaccine-antigen directed CD4 and CD8 T cells as well as regulatory T cells (T Regs). One stage IV patient showed a complete response of lung metastases, and after a relapse that was surgically treated remains tumor free until today [10].

RNActive® vaccines are novel, mRNA-based vaccines containing both free and protamine-complexed mRNA. They support optimal expression of the encoded antigen as well as innate immune stimulation with a built-in adjuvanticity that is at least partly mediated via Toll-like receptor 7 activation [11, 12]. In mice, immunization with these self-adjuvanted vaccines leads to a boostable and balanced humoral as well as T cell-mediated antigen-specific immunity, which is long lived as shown by presence of antigen-specific memory T cells [13, 14].

CV9103 is such a self-adjuvanted mRNA vaccine targeting 4 antigens: prostate-specific antigen (PSA), prostate-specific membrane antigen (PSMA), prostate stem cell antigen (PSCA), and six-transmembrane epithelial antigen of the prostate 1 (STEAP1). In healthy men, these antigens are frequently and almost exclusively expressed in the prostate [15–18], and overexpressed in prostate cancer; with the exception of PSMA which is also overexpressed in the tumor-neovasculature of other cancers [15, 19]. After radical prostatectomy or radiation, these antigens are mainly found on residual prostate cancer cells [20–25]. In addition, all four antigens were shown to be immunogenic in humans, where they induce T and B cell responses [26–28].

CV9103 encodes full-length antigens and thus has the advantage to induce an immune response against all epitopes contained in the target protein without HLA-restrictions. This, and the inclusion of multiple antigens, was developed to reduce the risk of tumor immune escape due to loss of expression of individual antigens [28], to increase the clinical efficacy by inducing a broader immune response and to provide immune responses against antigens present in the individual tumor in a higher number of patients.

Here we report the results of a phase I/IIa study that evaluated the safety and tolerability of CV9103 following intradermal administration in patients with advanced CRPC. The induction of antigen-specific immune responses is also reported.

## Results

### Patient characteristics

Between January and November 2009, 50 patients with advanced CRPC were screened at 12 centers in Germany and Italy. 44 patients were eligible, started treatment with CV9103 and were included in the safety population. Of these, 12 patients were enrolled in the phase I part of the study and 32 patients in the phase II part. Of all patients, 40 (91 %) were evaluable for PSA response and 2 (5 %) for tumor response according to Response Evaluation Criteria in Solid Tumors (RECIST). At the highest dose level, 33 (86 %) patients were evaluable for immune response analysis. Patient baseline characteristics are depicted in Table 1. 36 patients had metastatic disease, including 26 patients evaluable for immune response.

**Table 1:** Baseline characteristics of the patient population (N = 44)

Characteristic	Total (N = 44)
Age, years	
Median	67
Range	51–84
Gleason score available, n (%)	39 (89)
≤7	15 (38)
>7	24 (62)
PSA level at baseline, ng/mL	
Median	22.2
Range	0.2–746
Extent of disease, n (%)	
Local relapse	6 (14)
Locoregional	1 (2)
Metastases total	36 (84)
Peritoneum	1 (2)
Pelvis	1 (2)
Bladder	1 (2)
Lung	3 (7)
Liver	1 (2)
Lymph nodes	26 (59)
Bone	28 (64)
ECOG, n (%)	
0	43 (98)
1	1 (2)
Time since diagnosis, weeks	
Mean	280.4
SD	228.15
Previous therapy, n (%)	
Prostatectomy	23 (52)
Radiotherapy	28 (64)
Hormone ablation	44 (100)

ECOG, Eastern Cooperative Oncology Group; PSA, prostate-specific antigen; SD, standard deviation

### Treatment

During the phase I part, 12 patients received 256 (n = 3), 640 (n = 3), or 1280 μg (n = 6) mRNA. In the phase IIa part, another 32 patients were enrolled to receive the recommended dose (RD) of 1280 μg mRNA defined in phase I. All 44 patients received at least 2 vaccinations, 31 patients (70 %) received complete treatment with 5 vaccinations (see Additional file 1: Table S5). 5 phase I patients (11 % of total patient population) had a change in dosing, including 3 patients with a dose escalation as permitted by the protocol (1 at 256 μg, 2 at 640 μg). Two patients of the highest dose group had dose reductions to 640 μg due to adverse events (AEs; urinary retention, flank pain, and vomiting). Reasons for premature treatment discontinuation were disease progression (n = 6), disease progression and possibly related serious adverse events (grade 3 urinary retention, n = 1; grade 3 urinary retention with hydronephrosis, n = 1), unrelated serious adverse events (n = 2), non-compliance (n = 1) and withdrawal of consent (n = 1). Within the subgroup of metastatic patients, 4 were treated in the phase I part at 256 μg (n = 2) and 640 μg (n = 2) and 32 in the phase IIa part at 1280 μg mRNA.

### Results of phase I (dose escalation)

At the 2 lower dose levels, none of the patients experienced a dose limiting toxicity (DLT). Of the first 3 patients enrolled to the highest dose level, 1 experienced a DLT (Common Terminology Criteria for Adverse Events [CTCAE] grade 3 urinary retention; possibly related) at the week 5 visit. After week 5, this patient experienced 2 additional grade 3 DLTs, namely flank pain and vomiting. Therefore, another 3 patients were enrolled at this dose level. Two of these patients experienced DLTs of grade 3 (urinary retention) after the week 5 visit and were thus not considered for the determination of the RD. Since urinary retention is a common symptom of prostate cancer progression, the dose of 1280 μg was considered safe and recommended for the phase IIa part of the trial by the DSMB.

### Safety results

Treatment-related AEs were experienced by 39 (89 %) patients, with a total of 282 related AEs reported. The most frequent treatment-related side effects were injection site erythema and injection site reaction in 27 (61 %) and 21 (48 %) patients, respectively. Fatigue (18 %), pyrexia (16 %), chills (11 %), and influenza-like illness (11 %) were also frequently reported (Table 2). A trend for a dose-dependency of these AEs was not observed. In general, AEs were manageable and resolved upon therapy.

**Table 2:** Adverse events considered related to study medication per dose group

Adverse event^1^ (SOC/PT), n^2^ (%)	Treatment group								
	256 μg mRNA		640 μg mRNA		1280 μg mRNA		Total N = 44		Total N = 44
	N = 3		N = 3		N = 38				
	Grade 1/2	Grade ≥3	Grade 1/2	Grade ≥3	Grade 1/2	Grade ≥3	Grade 1/2	Grade ≥3	All Grades
Total patients with related AEs	2 (67)	1 (33)	2 (67)	1 (33)	30 (79)	3 (8)	34 (77)	5 (11)	39 (89)
General disorders and administration site reactions	2 (67)	1 (33)	3 (100)	0	32 (84)	0	37 (84)	1 (2)*^1^	38 (86)
Injection site erythema	2 (67)	0	2 (67)	0	23 (61)	0	27 (61)	0	27 (61)
Injection site reaction	2 (67)	0	1 (33)	0	18 (47)	0	21 (48)	0	21 (48)
Fatigue	2 (67)	0	0	0	6 (16)	0	8 (18)	0	8 (18)
Pyrexia	0	0	1 (33)	0	6 (16)	0	7 (16)	0	7 (16)
Chills	1 (33)	0	0	0	4 (11)	0	5 (11)	0	5 (11)
Influenza-like symptoms	0	0	0	0	5 (13)	0	5 (11)	0	5 (11)
Musculoskeletal and connective tissue disorders^3^	1 (33)	0	0	0	5 (13)	1 (3)	6 (14)	1 (2) ^*2^	7 (16)
Nervous system disorders^4^	1 (33)	0	1 (33)	0	4 (11)	0	6 (14)	0	6 (14)
Renal and urinary disorders^5^	0	0	0	1 (33)	3 (8)	2 (5)	3 (7)	3 (7)^*3^	6 (14)
Skin and subcutaneous disorders^6^	0	0	0	0	6 (16)	0	6 (14)	0	6 (14)
Gastrointestinal disorders^7^	1 (33)	0	0	0	3 (8)	1 (3)	4 (9)	1 (2)^*4^	5 (11)

^1^ Adverse events (AEs) by system organ class (SOC) and/or preferred term (PT) that occurred in at least 10% of patients

^2^ Multiple occurrences of the same AE in one patient are counted once

^3^ Musculoskeletal and connective tissue disorders includes the following terms: arthralgia, muscle spasms, muscular weakness and pain events

^4^ Nervous system disorders includes the following terms: somnolence, tremor, disturbance in attention, dizziness, dysgeusia

^5^ Renal and urinary disorders includes the following terms: urinary retention, urinary tract obstruction, dysuria, hydronephrosis, micturition urgency, obstructive uropathy and urinary incontinence

^6^ Skin and subcutaneous tissue disorders includes the following terms: erythema, pruritus

^7^ Gastrointestinal disorders include the following terms: diarrhea, constipation, abdominal pain, dry mouth, gingival bleeding, vomiting

*^1^disease progression; *^2^flank pain; ^*3^urinary retention (n = 2); urinary retention and hydronephrosis (n = 1); *^4^vomiting

The majority of related AEs were of mild to moderate intensity. In 5 (11 %) patients, related AEs of CTCAE grade 3 were reported, including urinary retention (n = 3), vomiting (n = 1), disease progression (n = 1), decreased neutrophil count (n = 1), flank pain (n = 1), confusional state (n = 2), dyspareunia (n = 1), and hydronephrosis (n = 1). The decreased neutrophil count (800/μl with a total leucocyte count of 3900/μl) was measured only at the week 5 visit and neutrophils were again within normal range in week 7. The patient was otherwise asymptomatic. Life-threatening related AEs (CTCAE grade 4) were not observed. One fatal AE was reported for a 68-year old patient with bone metastases and a history of obstructive pulmonary disease and right-sided heart insufficiency. He was hospitalized due to disease progression and bronchopneumonia 1 month after having received the 4th vaccination. The event was considered possibly treatment related since a relationship to vaccination could not be excluded.

A total of 21 serious AEs (SAEs) were reported in 9 (20 %) patients, with the most frequent SAEs being urinary retention and anemia in 3 (7 %) patients each and hematuria in 2 (5 %) patients. SAEs considered possibly treatment-related were reported in 2 (5 %) of patients (grade 3 urinary retention and grade 3 urinary retention with hydronephrosis) and occurred after the second vaccination. The events resolved after symptomatic and antibiotic treatment. No adverse events indicating autoimmune reactions were reported.

### Immune responses

To define optimal methodology, cells from 3 patients at 1280 μg and 2 patients at 640 μg were re-stimulated *in vitro* in the presence of vaccination antigens and cytokines to increase the frequencies of vaccine-induced antigen-specific cells before analysis. However, to generate reliable results that reflect the physiological response, we decided to analyze the other immunological evaluable patients *ex vivo,* including 33 high dose patients.

In total, 38 of 44 patients were evaluable for immune responses according to study protocol. Criteria to define responders were adapted from CIMT/CIC criteria [29].

**Fig. 1:**
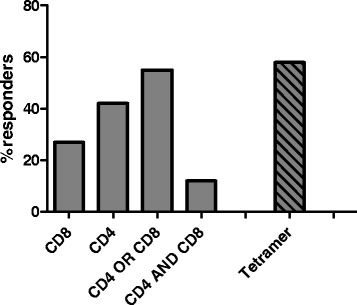
Cellular immune responses to CV9103. Evaluation of antigen-specific cellular immune responses as measured *ex vivo* by ELISpot, ICS and tetramer staining. Shown is percentage of patients responding against ≥ one antigen at ≥ one time point within evaluable patients (n = 33). Percentages of responding patients are indicated for patients eliciting a CD4 response, a CD8 response, both CD4 and CD8 responses and either CD4 or CD8 responses. Tetramer binding was measured only in HLA-A2+ patients (n = 14)

Quantitative analysis of ELISpot, ICS, and tetramer staining assays revealed that CV9103 was able to induce both CD4 and CD8 T cell responses. The percentage of high-dose patients with increased frequencies of T cells releasing cytokines in an antigen-specific manner after vaccination was 27 % for CD8 cells and 42 % for CD4 cells (Fig. 1). In these patients, numbers of antigen-specific cells against at least one antigen were increased compared with baseline at least at one time point. 12 % of patients showed both CD8 and CD4 responses, while 55 % of patients had either CD4 or CD8 responses as assessed by ELISpot and ICS.

In 12 HLA-A2-positive patients at 1280 μg, antigen-specific CD8 T cells were detected in 7/12 (58 %) of patients by tetramer staining (Fig. 1). Overall, in 25/33 (76 %) evaluable patients treated at 1280 μg a cellular immune response could be detected.

Analysis of humoral responses to vaccine antigens was limited in this study, because only PSA and PSCA were available as full proteins eligible for ELISA, and amounts of PSCA protein allowed for testing 23 patients at 1280 μg only. Although antibody levels were already detectable at baseline in almost all patients hinting at pre-existing humoral immune responses, 4 patients showed an increase of anti-PSA antibodies over the course of vaccination. No increase of anti-PSCA antibodies in evaluated patients could be detected.

**Fig. 2:**
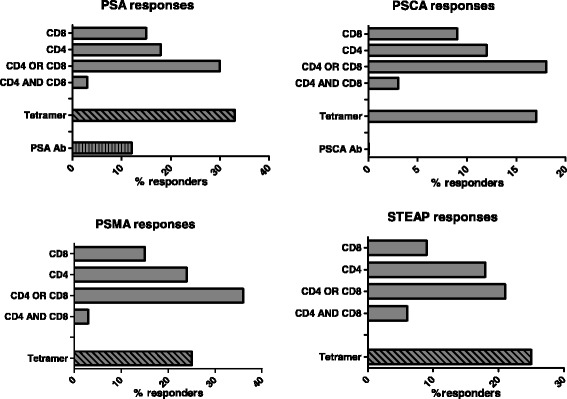
Evaluation of antigen-specific cellular and humoral immune responses per antigen. Percentage of patients (n = 33) responding against each antigen contained in the vaccine by CD4 response, CD8 response, both CD4 and CD8 responses and either CD4 or CD8 responses. Tetramer responses were measured only in HLA-A2+ patients (n = 14), humoral responses were measured only against PSA and PSCA and could only be detected for PSA, 23 patients tested for anti PSCA antibodies showed no increase over time

**Fig. 3:**
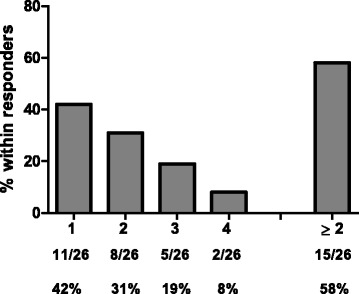
Responses against multiple antigens. Frequency of patients responding against multiple antigens within responding patients. Percentages of positive patients are indicated for the number of recognized tumor antigens and for multiple (≥2) recognized antigens in total. The majority of immune-responding patients exhibited responses against ≥2 different tumor antigens (PSA, PSCA, PSMA and STEAP1)

**Fig. 4:**
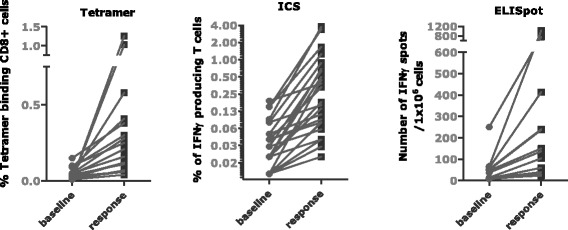
Antigen-specific T cells (frequency and number) at baseline and positive time point. Values are given in percentage of positive cells gated on CD8+ population for tetramer staining (n = 17), percentage of positive cells gated on CD4+ or CD8+ populations for intracellular cytokine (ICS) staining (n = 26) and SFC/1×10^6^ total PBMCs for IFN-γ ELISpot (n = 14). Responses are showing the maximum increase post-baseline compared with baseline irrespective of the time point

Of note, cellular CD4 and CD8 immune responses against all 4 antigens of CV9103 were observed (Fig. 2), independent of their cellular localization (PSA: secreted protein, PSCA: GPI-anchored protein, PSMA: type II trans-membrane protein, STEAP: six trans-membrane spanning protein, see Additional file 1: Table S1). 42 % of immunologically responding patients responded to only 1 antigen (single responders), whereas 31 % recognized two, 19 % three and 8 % all four antigens, resulting in 58 % multiple responders (patients responding against more than 1 antigen; Fig. 3). As expected for *ex vivo* assays, the absolute frequency of antigen-specific T cells is relatively low; notably, in the majority of responders, the T cells increased more than 2-fold compared with baseline (Fig. 4).

Within the observed range, immune responses after vaccination with CV9103 were not age-dependent. At the recommended dose, an overall response was detected in 75 % of patients ≤65 years (n = 12), whereas in patients above 65 years (n = 21), CV9103 vaccination showed an overall immunogenicity in 81 % of patients (Additional file 2: Figure S1). In addition, logistic regression analysis did not identify a significant correlation between age and immune responses (p = 0.88). These results are in line with preclinical experiments in mice, where newborn as well as old mice showed similar immune responses after RNActive® vaccination [30].

At 256 μg, none of the 3 patients treated showed an immune response to any of the included antigens. At 640 μg, immune responses were observed in 2 patients, including 1 patient with a humoral response to PSA and 1 patient with a cellular response to PSA, PSMA and STEAP after *in vitro* re-stimulation. These patients were not included in the overall analysis of immune responses.

To evaluate frequencies of lymphocyte subpopulations and expression of activation and maturation markers, we performed cell phenotyping including antibody panels specific for B cells, T cells, NK cells and regulatory T cells. A striking decrease or increase as compared to baseline levels of any lymphocyte subset or maturation or activation markers on those subsets was not observed. However, the frequency of antigen-unspecific CD19+ B cells was increased in 68 % of patients at two or three time points after vaccination, with at least two-fold frequencies compared with baseline in 14/31 patients (45 %) (Additional file 2: Figure S2). For a summary of immune responses see Additional file 1: Tables S4, S6, S7).

### Clinical efficacy results

To assess a potential impact of CV9103 on the clinical course of prostate carcinoma patients, PSA progression-free survival (PSA-PFS; interval from the first vaccination to PSA progression according to Prostate Cancer Working Group 2 [PCWG2] criteria) was measured in 38 patients at 1280 μg CV9103. Median PSA PFS was 1.8 month [95 % CI: 1.4; 3.2], the 6 months PSA PFS rate was 15.9 %.

One patient with lymph node metastases and a Gleason score of 9 at diagnosis had a confirmed PSA response with a maximum decline of PSA >80 % from baseline in week 23 without any supplementary anti-cancer therapy (Additional file 3 shows this case in detail). After an initial rise in week 7, a substantial decline of PSA level was observed in this patient, which was confirmed another 4 weeks later (Baseline PSA: 3.96 μG/L; Week 7: 9.75 μG/L, Week 23: 1,36 μG/L, confirmed one month later: 0,96 μG/L). No further PSA follow up values were available. The patient died 30 months after start of CV9103 treatment. Immunologically, this patient had a cellular immune response to PSA with a 9-fold increase of PSA-specific CD4 T cells detected by ICS in week 9. A positive humoral response against PSA according to pre-defined criteria was not detected in this patient due to high assay variation at baseline, still levels of anti PSA IgG exceeded the baseline levels at week 5.

In 2 patients, tumor assessment according to RECIST was evaluable, but none of them showed an objective response.

**Fig. 5:**
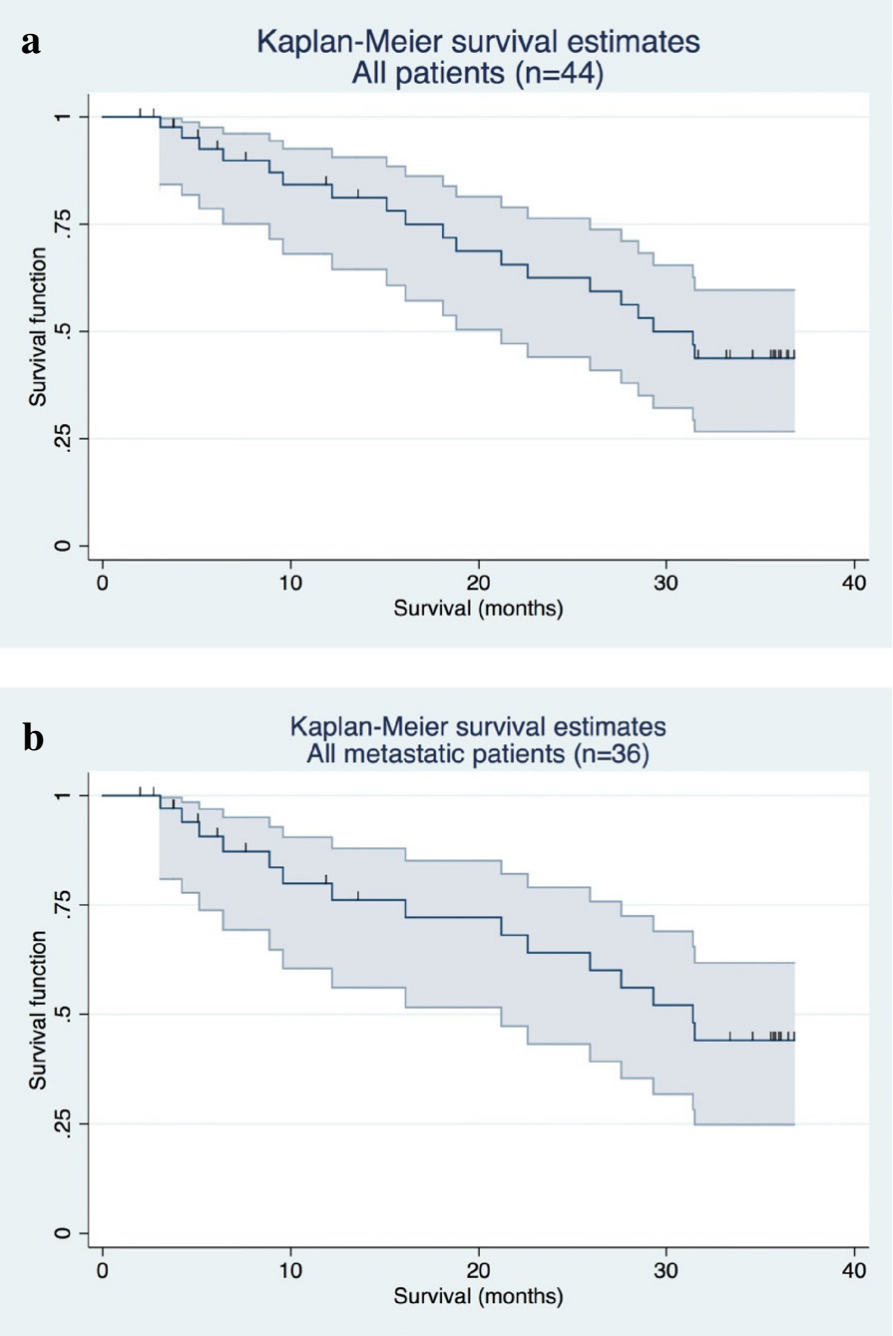
Kaplan-Meier plots of overall survival. Kaplan-Meier survival estimates are presented for (**a**) All patients (n = 44): median OS was 29.3 months 95 % CI: [21.2; n.a]. (**b**) Patients with metastatic disease (n = 36): median OS was 31.4 months 95 % CI: [21.2; n.a.]

Median OS for all 44 patients was 29.3 months [95 % CI: 21.2; n.a.] (Fig. 5a), in the subgroup of patients with metastatic disease (n = 36), median OS was 31.4 months [95 % CI: 21.2; n.a.] (Fig. 5b).

### Correlation of immune response and survival data

**Fig. 6:**
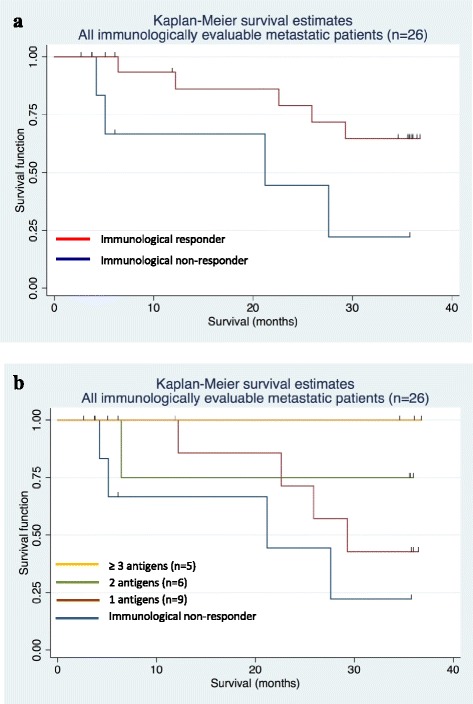
Kaplan-Meier plots of overall survival by immunological responses. (**a**) Kaplan-Meier survival curves of metastatic patients evaluable for immune responses (n = 26), including immunological responders (n = 20) and non-responders (n = 6). The Hazard Ratio of survival for responders versus non-responders was 0.30, [95 % CI: 0.08; 1.15], p = 0.09. (**b**) Kaplan-Meier survival curves of metastatic patients evaluable for immune responses (n = 26), evaluated according to the number of CV9103 antigens the patients responded to. The Hazard Ratio was 0.41, [95 % CI: 0.17; 0.95], and p = 0.017

To further assess the clinical impact of CV9103, the effect of immune responses on survival was analyzed. 33 patients were immunologically evaluable. Of these, 26 patients had metastatic disease, including 20 patients (77 %) with cellular immune responses as analyzed by *ex vivo* methods and 1 patient (within these 20 patients) showing an additional humoral response. Correlation of current survival data with observed immune responses after treatment with CV9103 revealed a trend for longer survival times in patients responding to the vaccine (Fig. 6a). The HR for death of these patients compared with immunological non-responders was 0.3 [95 % CI: 0.08; 1.15] with a p-value of 0.09 (Fig. 6a).

Furthermore, patients exhibiting an immunological response to more than one antigen (multiple responders) had longer survival times than non-responding patients or patients responding to only one antigen (HR = 0.41, [95 % CI: 0.17; 0.95], p = 0.017). Kaplan-Meier survival estimates were highest in patients responding to 3 or 4 antigens of CV9103 (Fig. 6b).

## Discussion

For men with metastatic CRPC, treatment options have been limited in the past. In the recent years though, a range of novel treatments, including several immunotherapy approaches, have been in development. Here, we report the tolerability, safety and immunogenicity of a novel immunotherapy for patients with advanced CRPC using recently developed modified mRNA molecules as a vaccine. CV9103, encoding the full length proteins PSA, PSCA, PSMA and STEAP, was designed and tested in a phase I/IIa clinical study. In phase I, 3 doses of CV9103 were administered to patients to define a safe dose for phase IIa. The highest dose of 1280 μg mRNA was selected for the extension cohort.

CV9103 mRNA was well tolerated and showed a good safety profile. The observed cases of urinary retention and hydronephrosis reported in the present trial might have been related to progression of the underlying prostate cancer, with tumor masses impacting the urethra or the ureters. However, a vaccine-induced immune response may stimulate infiltration of lymphocytes into the tumor, which could result in an increased tumor volume representing a pseudo-progression, as reported for other immunotherapies [31]. Hence, a potential contribution of CV9103 to these events cannot be excluded, and might have been associated with the desired immune reaction against the tumor. In future trials, tumor biopsies taken at the time of tumor volume increase may help to clarify the etiology of such events.

The observed injection site erythema, injection site reactions and influenza-like symptoms were mild to moderate and have been observed in previous clinical trials with cancer vaccines [5], including mRNA vaccines [10, 32, 33]. Serious AEs of autoimmunity have been reported in recent trials with the checkpoint inhibitors ipilimumab (anti-CTLA-4 monoclonal antibody) and nivolimumab (anti-PD1 monoclonal antibody) [34–36], and also rarely for other cancer vaccines. Importantly, we did not observe any clinical AEs indicative of autoimmune reactions upon administration of CV9103.

In addition to a good tolerability and safety profile, CV9103 was shown to be immunogenic in the present study. To induce an effective immune attack against cancer antigens it is necessary to overcome immunological tolerance [37] by combining immunogenic antigens with a strong adjuvant. The new format RNActive® carries both the antigen and the necessary adjuvant effect within one vaccine. Accordingly, the present data on immunogenicity of CV9103 showed that immune responses could be elicited against all encoded antigens, independent of their cellular localization (Additional file 1: Table S1). In order to measure physiological numbers of vaccine-induced antigen-specific cells, PBMCs were not re-stimulated *in vitro* with the vaccination antigens prior to conducting the assays. A ≥2-fold increase of cytokine releasing cells after antigenic stimulation as measured by *ex vivo* ELISpot or ICS was observed in 19/33 (58 %) of patients at the recommended dose. This antigen-specific immunity consisted of both CD4 and CD8 T cells. 7 out of 12 HLA-A2 positive patients had increased frequencies of MHC-multimer-binding cells after vaccination. Hence, a cellular immune response was observed in 76 % (25/33) of the patients, in some as early as after the second immunization. The observed immune responses were not consistent over time in the majority of patients, a similar kinetics has also been seen in other cancer vaccine trials[38]. In the described study, possible reasons for this may be the low number of sampling timepoints, the timing of blood sampling in relation to vaccination timepoints, or the detection limit for immunological *ex vivo* assays.

It cannot be excluded that the results of the present study underestimate the true level of T cell responses induced by vaccination with CV9103. The immunogenic epitopes derived from the full-length mRNA of CV9103 are HLA-genotype-dependent and hence may vary in between patients. Analyses of T cell responses were conducted using selected peptides of the 4 antigens, either previously described or predicted, which may not represent all epitopes generated in every patient. In addition, analyses were performed *ex vivo* to obtain physiologically realistic results regarding frequency of induced immune responses. After initial testing in few patients, an *in vitro* re-stimulation was not performed to prevent artificial generation of antigen-specific T cells in cell culture. Regarding the limits of detection of the applied assays, this may render an explanation for inconsistent responses in different assays.

The assessment of humoral immune responses was restricted to PSA and PSCA, since no proteins suitable for ELISA were available for the other vaccination antigens. An increase of PSA-specific antibodies could be detected in 4/33 patients, but no increase of anti-PSCA antibodies was observed. To our knowledge, induction of PSCA-specific antibodies has not been investigated so far, although PSCA has been used as a target for immunotherapy of prostate cancer using various vectors [39].

Of note, 58 % (15/26) of responding patients and 45 % (15/33) of all evaluable patients at the highest dose level showed responses to multiple vaccination antigens (31 % against two, 19 % against three, 8 % against all four antigens). Similar overall immunological results were obtained in previous clinical trials with non-RNActive® mRNA vaccines [10, 32, 33, 40]. Recent data published on a peptide-based vaccination approach in renal cell carcinoma suggested that multi-antigen responses may be related to improved survival [38]. A trend towards longer survival times was also observed in patients responding to more than one antigen of CV9103.

The frequency of regulatory T cells was assessed using an epigenetic assay that is based on a PCR-assay measuring the methylation level of the FoxP3 gene and is described to detect regulatory T cells from peripheral blood more reliably than classical CD4^+^CD25^hi^FoxP3^+^ intracellular staining [41]. During the course of vaccination, no change in the frequency of regulatory T cells could be detected in any of the patients. This result is in line with pre-clinical experiments, where no de- or increase of CD4^+^FoxP3^hi^ regulatory T cells was detected, while antigen specific CD4 and CD8 T cells as well as tumor responses were measured. Also, no relation between frequency of regulatory T cells and overall survival time or regulatory T cells and immune response could be detected (Additional file 2: Figure S3).

Clinical efficacy of CV9103 was assessed mainly by progression of PSA, radiographic PFS was not assessed in this trial. Only two patients were evaluable for RECIST response evaluation since regular CT scans were not requested per protocol. Besides the small sample size and lack of a control group, the fact that no systematic tumor imaging was performed limits our ability to conclude on clinical efficacy. A median time to PSA-PFS of 1.8 months and an objective PSA response in only one patient upon therapy with CV9103 are typical results for cancer vaccines known to induce clinical response patterns that differ from those of cytotoxic agents and many targeted therapies. Cancer vaccines may e.g. induce delayed responses not seen in the first few months of therapy, or initiate a dynamic immune response that in spite of no observable tumor shrinkage will ultimately slow tumor growth rate [42]. In clinical trials with the prostate cancer vaccines Sipuleucel-T and ProstVac-VF, no significant effect on the time to objective disease progression was observed, although overall and 3-year survival was significantly increased [4, 5]. Importantly, PSA responses were also very rarely observed in these trials, indicating that PSA is no suitable surrogate to predict response to a cancer vaccine. Possibly, the effect of subsequent therapies may be increased by induced immune responses, resulting in a combination that is more effective than conventional treatments alone and may explain the prolonged survival despite lack of PFS prolongation. Indeed, patients that progressed on Sipuleucel-T and received docetaxel as subsequent therapy had much better overall survival than patients on placebo followed by docetaxel [43, 44]. In the subgroup of 36 patients with metastatic CRPC treated with CV9103, the Kaplan-Meier estimate of median OS was 31.4 months [95 % CI: 21.2; n.a.]. A non-significant correlation of survival time and immune responses was observed (HR = 0.30, p = 0.09). Outcome further improved in patients with responses to more than one antigen of CV9103 (HR = 0.41, p = 0,017). The analysis of immunological response as a predictor for survival was restricted to simple univariate models treating the immunological response as baseline variable. Due to low sample size the presented analysis is regarded to be an adequate presentation of the underlying data. A correlation of immune responses against more than one antigen and improved survival time has also been shown by others [38] and does not necessarily imply a therapeutic effect of vaccination since the ability to mount an immune responses after vaccination might be a surrogate of an improved prognosis.

Thus the investigation of the vaccine in a controlled clinical trial seems justified. Based on these results, a randomized, placebo-controlled phase IIb study in CRPC started to enroll patients in 2012 using CV9104, an advanced vaccine variant of CV9103 (www.clinicaltrials.gov, NCT01817738). The primary endpoint of this trial is OS with PFS according to the criteria of the PCa working group as secondary endpoint. Further secondary endpoints will clarify the impact of immune response on overall survival and seek to determine biomarkers associated with improved survival. 197 patients with metastatic asymptomatic or minimally symptomatic CRPC have been randomized, a population expected to benefit from vaccinations as shown in the past. By using a large sample size and a placebo group, the trial will allow for evaluation of the therapeutic potential of mRNA vaccination against multiple antigens in PCa.

Interestingly, combinations with other immuno-modulatory agents might contribute to breaking tumor tolerance [45] and thus further improve novel, immunetherapeutical approaches such as treatment with self-adjuvanted mRNA molecules. Pre-clinical experiments in mouse models have indeed shown a synergistic effect of combination regimens including mRNA vaccination with CTLA-4 blockade [13] or radiotherapy (unpublished data), suggesting that the investigation of these combinations in clinical trials might be promising.

## Conclusions

In conclusion, in the present study, vaccination by intradermal injection of CV9103, a self-adjuvanted, two-component, full-length mRNA vaccine encoding several tumor associated antigens of PCa, was well tolerated and induced immune responses that may result in prolonged survival in patients with CRPC. Overall, this trial suggests that the RNActive® vaccination technology constitutes an effective and nimble platform to generate immune responses against virtually any protein antigen.

## Methods

### RNActive® technology

CureVac GmbH proprietary technology generated mRNA molecules with increased stability and translatability (patents EP1392341, EP1857122, and application WO2012019780A1). The RNActive® vaccines consisted of a mixture of free modified mRNA (component 1) and mRNA complexed with protamine at a weight ratio of 2:1 (component 2). First, mRNA was complexed by the addition of protamine-Ringer lactate solution and, after stable complexation, free mRNA was added [14]. All mRNA vaccines used in the present study were produced in accordance with current Good Manufacturing Practice guidelines.

### Patients

Study participants were at least 18 years of age with progressive metastatic or non-metastatic CRPC, defined by a rise in PSA at three consecutive time points (PSA rise over nadir, separated by >1 week, PCWG2 criteria) and/or RECIST-based or bone-scan based progression of evaluable lesions while the patient has a castrated level of testosterone (achieved either by orchiectomy or GNRH analogue with or without anti-androgen). Anti-androgens had to be discontinued at least 4 weeks prior to enrollment to exclude a withdrawal response. Patients had to have a life expectancy of >12 months as assessed by the investigator. Previous chemotherapy was not allowed and patients with a history of autoimmune disorders were excluded. Eligibility criteria included an Eastern Cooperative Oncology Group (ECOG) performance status of ≤1 and adequate total bilirubin and renal, hepatic, cardiac, and bone marrow function. Patients requiring bisphosphonates at the time of registration into the trial were eligible as long as therapy was initiated at least 28 days prior to first study treatment administration and continued at a constant level during the study period (for a complete list of eligibility criteria see Additional file 1: Table S2).

### Study design and treatment

The present study was a prospective, multicenter, open-label, uncontrolled phase I/IIa trial performed at 12 study sites in Germany and Italy (for list of involved ethic commitees see Additional file 4: Table S8). The starting dose of 256 μg mRNA was selected based on data of single and repeat dose toxicity studies in mice. In these toxicity studies, a No Observed Adverse Effect Level (NOAEL) was not reached up to an mRNA dose of 256 μg per application. Dose escalation of CV9103 (phase I) was based on a Fibonacci 3 + 3 design with dose-limiting toxicity (DLT) defined as Common Terminology Criteria of Adverse Events (CTCAE) grade 3/4 related neutropenia with fever and/or infection, CTCAE grade 3/4 related non-hematological toxicity, or dosing delay >2 weeks due to toxicity. Primary objective of the phase I part was determination of the recommended dose (RD) for phase II, defined as the highest dose level in which DLT was observed in ≤1 of 6 evaluable patients before the Week 5 visit. In the extension cohort at RD (phase IIa), the primary objective was assessment of safety. Secondary objectives were evaluation of induction of immune responses and anti-tumor activity. Immunological efficacy endpoints were the assessment of induction of antigen-specific cellular and humoral immune responses and the evaluation of levels of regulatory T cells during the course of treatment. Clinical efficacy endpoints were PSA-PFS, objective response according to RECIST and overall survival. Radiographic imaging was to be performed at the discretion of the investigator.

Immuno-suppressants, systemic steroids as well as anticancer agents or investigational agents were prohibited during the course of the study treatment. Patients experiencing pain or fever following vaccination could be treated with non-steroidal anti-inflammatory drugs such as ibuprofen as required.

CV9103 consists of RNActive®-based mRNA components encoding the antigens PSA, PSMA, PSCA, and STEAP1 (Additional file 1: Table S1). At every vaccination, 64 μg, 160 μg, or 320 μg mRNA of each antigen was administered for a total dose of 256 μg, 640 μg, or 1280 μg mRNA, respectively. Each CV9103 component was administered as 2 intradermal injections of 200 μl each (total: 8 injections per vaccination), one into the thigh and one into the upper arm of the same body half. Injection sites of each antigen were rotated clockwise at different vaccination days. Patients were vaccinated on weeks 1, 3, 7, 15, and 23. After the last vaccination further anti-tumor therapy was at the discretion of the investigator. Data cutoff for survival analysis was December 2012.

### Assessment of safety

Adverse events (AEs) were recorded at each visit and graded according to the National Cancer Institute (NCI) Common Terminology Criteria of Adverse Events (CTCAE) version 3.0 from the first dose until 28 days after the last dose of CV9103. Relatedness to vaccination was assessed by the investigators and events that were considered at least possibly related were counted as related in the final analysis. During dose-escalation, DLT occurring prior to Week-5 visit was assessed. The escalation to higher dose levels and the selection of the RD took place under guidance of a Data Safety Monitoring Board (DSMB) consisting of investigators, the sponsor’s medical representative, the CROs medical monitor and an independent expert. Routine hematological and biochemical tests were performed at all visits and autoimmunity assessments (rheumatoid factor, antinuclear antibody titer, TSH, antibodies against thyroglobulin and smooth muscle antibodies) were assessed at baseline, week 5, 15, and at the end of the study.

### Assessment of immunogenicity

Immune responses were evaluated in all patients who received at least 3 vaccinations. The rate of vaccine antigen-specific humoral and cellular immune responses as well as regulatory T cell frequency was determined at weeks 5, 9, and 17 (2 weeks after 2nd, 3rd and 4th vaccination) and compared to baseline. Humoral immune response was assessed by IgG- or IgM-ELISA. Cellular immune response (antigen-specific T lymphocytes) was assessed by ELISpot, ICS, and tetramer staining (see below). Phenotyping was performed to identify various lymphocyte subsets and maturation/activation markers on NK, B and T cell subsets*.* For analysis, samples of patients were thawed and all time points from each patient were analyzed simultaneously. All laboratory procedures were performed according to approved, validated SOPs and in the style of CIC/CIMT protocols [29]. ELISpot, ICS and Tetramer staining were validated using a model antigen (CMV) for inter- and intra assay precision, specificity, robustness and linearity (ELISpot only) according to ICH guidelines. A patient was considered to be a responder if they responded in at least one assay against at least one antigen at least one time point.

### Preparation of peripheral blood mononuclear cells (PBMCs)

PBMC preparation from blood samples was performed using a study specific PBMC preparation kit (Interlab GmbH, Munich, Germany) containing all necessary materials and buffers. Briefly, blood was transferred from the heparin tube to the Leucosep tube (Greiner Bio-One, Germany) and centrifuged. PBMCs were harvested from the interphase and washed twice. Viability was assessed by Trypan-Blue staining, yield and red blood cell contamination was assessed by Turk’s solution for cell counting. Cells were frozen till analysis in DMSO-free freezing medium (Cryo-SFM, Promocell).

### Enzyme-linked immunosorbent assay (ELISA)

The levels of anti-PSA and anti-PSCA antibodies (IgG and IgM) were evaluated by ELISA using four serial dilutions. The wells of a maxisorb plate were coated with purified human PSA protein (Scipac), or recombinant PSCA protein (CCS, Hamburg, Germany). After blocking, patient plasma was added. A secondary antibody (anti-IgG-HRP and anti-IgM-HRP, Jackson ImmunoResearch) was used for detection by the ELISA reader (Tecan Sunrise, Biotek Synergy HT) (absorbance at 450 nm). Serum of healthy volunteers was used as negative control, antigen-specific monoclonal mouse-anti-human antibodies were used as control. Antibody responses were considered positive if samples were ≥ baseline value (Week 1) plus 2 times standard deviation of baseline titer (S.D.).

### Epitope selection

As target in cellular cytokine secretion assays, patient specific peptide cocktails were used. Predicted peptide pools for each antigen consisted of epitopes for the following HLA-types: Class I: HLA-A1, HLA-A2, HLA-A3, HLA-A24, HLA-B7, HLA-B8, HLA-B35, HLA-B44 and Class II: HLA-DRB1-01, HLA-DRB1-03, HLA-DRB1-04, HLA-DRB1-07, HLA-DRB1-011, HLA-DRB1-015, and were chosen according to the patient’s haplotype.

Preferably, known immunogenic epitopes were used. For antigens or haplotypes with no known epitopes, a binding prediction with SYFPEITHI (http://www.syfpeithi.de), Rankmap, Bimas (http://www-bimas.cit.nih.gov/molbio/hla_bind/) and Net MHC (www.cbs.dtu.dk/services/NetMHC/) was performed. Altogether, 2–3 peptides per allele (2–9 per antigen) were used (Additional file 1: Table S3).

### Enzyme-linked immunosorbent spot assay (ELISpot)

According to CIMT/CVC protocol recommendations [29, 46–48] PBMCs were rested overnight at 37 °C and 5 % CO_2_ in cell culture medium and seeded at 300.000 cells/well in 200 μL serum-free culture medium in 96-well PVDF plates pre-coated with anti-IFN-γ antibody. After incubation for 24 hours, cells were stimulated with serum-free X-Vivo 15 medium (Lonza) containing individual peptide pools as described above. PBMCs cultured in the presence of Staphylococcus enterotoxin B (SEB) and CMV/Eppstein-Barr/Flu (CEF) peptide mix (JPT) were used as control for cell reactivity. The number of IFN-γ-producing cells was evaluated using an automated ELISpot reader (CTL ImmunoSpot Analyzer, Cellular technology limited). Antigen-specific T cell responses were considered positive if all of the following five criteria were fulfilled: samples were ≥2-fold background value (negative control peptide) and ≥2-fold baseline value (Week 1), displayed a minimum of 5 spots and could be measured as triplicate values (at least duplicates in case of limited cell numbers), and were without overlapping error bars (adapted from: response definition criteria for ELISpot assays, Association for Cancer Immunotherapy (CIMT)). Representative data for ELISpot and other assays used to monitor immune responses is shown in Additional file 2 (Figure S4).

### Intracellular cytokine staining (ICS)

Two million PBMCs were incubated at 37 °C with antigenic peptides pools of PSA, PSMA, PSCA, and STEAP1 in the presence of anti-CD28 antibody for 1–1.5 h and, after addition of Brefeldin A and Monensin, for additional 6-8 h. For flow-cytometric analysis, the cells were stained with a viability dye to exclude dead cells, followed by staining of CD4, CD8 and CD56. After permeabilization, staining for IFN-γ and TNF-α was performed. The proportion of cytokine-producing cells within the CD8+ and CD4+ T cell populations was calculated. Antigen-specific T cell responses were considered positive, if samples were ≥2-fold background (negative control peptide) and ≥2-fold baseline value (Week 1) and had at least 0.02 % positive cells.

### Tetramer staining

Two million PBMCs of HLA-A2-positive patients were stained with anti-CD4 and anti-CD8 antibodies and incubated with Streptavidin-PE- or Streptavidin-APC-conjugated tetramers of PSA, PSMA, PSCA, and STEAP1. Two tetramers were used for each antigen (monomers provided by Immatics Biotechnologies GmbH, Tuebingen, Germany). A tetramer containing an HLA-A2 peptide of human immunodeficiency virus (HIV) was used as a control. The proportion of tetramer-positive cells within the CD8+ T cell population was calculated. Antigen-specific T cell responses were considered positive if samples were ≥ mean negative control plus 3 times S.D. and ≥2-fold baseline value (Week 1) and had at least 0.02 % positive cells.

### Phenotyping

After thawing, 200.000 PBMCs were directly stained for B cell (CD19, CD25, CD69, CD80, CD86), T cell (CD3, CD4, CCR7, CCR5, CD25, CD69), and NK-cell markers (CD16, CD25, CD56, CD69) and analyzed by flow cytometry.

### Regulatory T cell measurement

Frequency of regulatory T cells was measured using a quantitative DNA methylation analysis of FOXP3. 1×10^6^ cells were frozen in 400 μl PBS and analyzed by Epiontis, Berlin (http://www.epiontis.com).

### Assessment of efficacy

Serum PSA levels were to be determined at baseline, at weeks 7, 15, and 23, at end of treatment, and every 3 months during follow-up until week 52 (follow-up assessments to be performed at local laboratories). Values obtained after start of subsequent therapies with potential effects on PSA levels (new anti-hormonal agents, corticosteroids, and/or chemotherapy) were not included in the assessment of PSA response. Patients who received vaccinations up to week 15 and had PSA assessments at least at baseline, week 7 and week 15 were evaluable for PSA response. PSA response and progression were assessed according to the PCWG2 criteria [31].

Radiological disease assessments were to be performed according to standard practice of the treatment center. Tumor response was assessed according to the Response Evaluation Criteria in Solid Tumors (RECIST) version 1.0 [49]. Patients with measurable disease according to RECIST who underwent a disease assessment within 1 month prior to treatment initiation and at least once during treatment were evaluable for RECIST response.

All patients were followed-up for survival for 3 years from start of the study. The frequency of assessment was every 3 months during the first year and every 6 months for 2 years after the week 52 visit.

### Statistical analysis

The sample size was chosen primarily on clinical and safety considerations, formal power calculations were not performed. The maximum number of enrolled patients was dependent on the number of DLTs and patients with early discontinuation of treatment. Dose escalation was based on a Fibonacci 3 + 3 design. The statistical analysis was descriptive. Means, medians, standard deviations, and ranges were provided for continuous outcomes while frequencies and percentages were reported for categorical data. Overall survival was estimated according to the Kaplan-Meier method. The relationships of explanatory variables to overall survival were analyzed by univariate Cox models using stratified by HLA-2+ type (due to additional Tetramer analysis in HLA-A2+ patients) Likelihood Ratio tests. For paired survival data (comparison with predicted survival) the sign-rank test was used. Overall survival was defined as the interval from the date of first vaccination to the date of death.

A retrospective analysis to evaluate the correlation between survival and induction of specific immune responses was performed by applying a landmark analysis at 17 weeks for the subgroup of 26 patients with metastatic disease who were evaluable for immune response assessment.

All analyses were carried out using Stata 13. All presented p-values are two-sided. The study was conducted in accordance with the Declaration of Helsinki and Good Clinical Practice guidelines. The protocol was approved by the ethics committees of participating study sites. Informed consent was obtained from all patients prior to any study-related procedures.

## Additional files

Additional file 1: Table S1. CV9103 antigens PSA, PSMA, PSCA and STEAP1. **Table S2.** Eligibility criteria. **Table S3.** Selected epitopes of CV9103 antigens. **Table S4.** Summary of all immunologically evaluable patients. Response against antigen, type of detected response, time of detected response and fold increase above baseline are summarized. **Table S5.** Number of subjects receiving 1, 2, 3, 4, or 5 vaccinations per cohort. **Table S6.** Antigen-specific responses for patients responding to only one antigen. **Table S7.** Antigen-specific responses for patients responding to two or more antigens. Additional file 2: Figure S1. Immune responses by age category (<65 or ≥65 years old) showing that immune responses were independent of age. **Figure S2.** CD19+ B cell levels showing anincrease over time. **Figure S3.** Analysis of regulatory T cells. No change in frequencies of regulatory T cells was seen during the course of vaccination and no difference between responders and non-responders was seen. **Figure S4.** Example assay data. Additional file 3:
**Case report of a 59 year old patient who developed a confirmed PSA response after initial increase in PSA.**
Additional file 4: Table S8. List of Ethical Bodies.

## Abbrevations

AE: Adverse event
CD: Cluster of differentiation
CRPC: Castration-resistant prostate cancer
CTCAE: Common Terminology Criteria for Adverse Events
DLT: Dose limiting toxicity
DNA: Deoxyribonucleic acid
ECOG: Eastern Cooperative Oncology Group
HR: Hazard ratio
PAP: Prostatic acid phosphatase
PCa: Prostate cancer
PCWG2: Prostate cancer working group 2
PFS: Progression free survival
PSA: Prostate specific antigen
PSCA: Prostate stem cell antigen
PSMA: Prostate specific membrane antigen
RD: Recommended dose
RECIST: Response Evaluation Criteria in Solid Tumors
RNA: Ribonucleic acid
SAE: Serious adverse event
SR: Survival rate
STEAP: Six-transmembrane epithelial antigen of the prostate
T Reg: Regulatory T-cell
