# Supplementary material for: Self-adjuvanted mRNA vaccination in advanced prostate cancer patients: a first-in-man phase I/IIa study
Source: J Immunother Cancer. 2015 Jun 16;3:26. doi: 10.1186/s40425-015-0068-y (PMC4468959; doi:10.1186/s40425-015-0068-y)
Supplement: Additional file 1: Table S1. — CV9103 antigens PSA, PSMA, PSCA and STEAP1. Table S2. Eligibility criteria. Table S3. Selected epitopes of CV9103 antigens. Table S4. Summary of all immunologically evaluable patients. Response against antigen, type of detected response, time of detected response and fold increase above baseline are summarized. Table S5. Number of subjects receiving 1, 2, 3, 4, or 5 vaccinations per cohort. Table S6. Antigen-specific responses for patients responding to only one antigen. Table S7. Antigen-specific responses for patients responding to two or more antigens. [file 40425_2015_68_MOESM1_ESM.pdf]

Table S1: CV9103 antigens PSA, PSMA, PSCA and STEAP1

**Antigen**	**Full name**	**Function**	**Localization**
PSA	Prostate-specific antigen (KLK3, kallikrein-related peptidase 3)	Serine protease	Extracellular
PSCA	Prostate stem cell antigen	Unknown	Cell surface, anchored by a GPI linkage
PSMA	Prostate-specific membrane antigen (FOLH1, folate hydrolase)	Glutamate carboxypeptidase	Transmembrane glycoprotein (Type II)
STEAP1	Six-transmembrane epithelial antigen of the prostate 1	Intercellular communication	Transmembrane glycoprotein (Type I)

GPI, glycosylphosphatidylinositol

Table S2: Eligibility criteria

Inclusion criteria	Exclusion criteria
• Signed informed consent in accordance with GCP and local regulatory requirements prior to trial participation<br>• Male and age ≥18 years<br>• Histologically confirmed diagnosis of adenocarcinoma of the prostate, Gleason score available<br>• Patients must have been treated with hormonal therapy and may have been treated with surgery and/or radiation therapy (see exclusion criteria)<br>• Progressive disease as defined by hormone refractoriness and rise in PSA. Hormone refractoriness: defined by rise in PSA, and/or RECIST-based progression of evaluable lesions, and/or increased number of hotspots on a bone scan, while the patient has a castrated level of testosterone. This castrated level may have been obtained by orchiectomy or LH-RH analog ± AA. AA must be discontinued for ≥4 weeks before study entry to exclude a withdrawal effect. Rise in PSA: rise in PSA level on 3 consecutive time points (PSA rise over nadir, separated by >1 week, PCWG2 criteria)<br>• Presence of metastatic disease is acceptable<br>• ECOG performance status of 0 to 1<br>• Life expectancy >12 months<br>• Adequate organ function<br>• Bone marrow function: hemoglobin ≥10 mg/dL; leukocytes ≥3,000/µL; lymphocytes ≥1,000/µL; absolute neutrophil count ≥1,500/µL; platelet count	• Other histologic type of prostate cancer (transitional cell, small cell, or squamous cell cancer)<br>• Symptomatic brain metastasis or leptomeningeal involvement<br>• Patient receiving systemic anticancer therapy<br>• Symptomatic congestive heart failure (NYHA 3 and 4); unstable angina pectoris; significant cardiac arrhythmia<br>• Pulmonary disease causing dyspnea or fatigue during normal activity; active significant infection<br>• History of seizures, encephalitis, or multiple sclerosis<br>• Documented history of active autoimmune disorders requiring systemic immunosuppressive therapy (e.g. sarcoidosis, lupus erythematosus, rheumatoid arthritis, glomerulonephritis, or systemic vasculitis), excepting autoimmune thyroiditis with only thyroid hormone replacement and stable disease >1 year inflammatory bowel disease e.g. Crohn's disease or ulcerative colitis; active diverticulitis<br>• Primary or secondary immune deficiency<br>• History of allergy requiring medication<br>• Active drug abuse or chronic alcoholism<br>• Clinically significant active infections<br>• Seropositive for HIV, HBV, or HCV

≥100,000/μL<br>• Renal: creatinine ≤1.5 mg/dL or creatinine clearance ≥60 mL/minepatic: AST and ALT ≤2.5 times ULN; bilirubin ≤1.5 ULN; HBV and HCV negative<br>• Concomitant LH-RH therapy continuation is acceptable<br>• May have had local palliative radiotherapy for bone metastasis involving <25% of bone marrow<br>• Patients requiring bisphosphonates at the time of registration into the trial are eligible (therapy initiated ≥28 days prior to first study treatment administration) and must be continued at a constant level during the study period<br>• Patients of child-producing potential must agree to use contraception while enrolled in the study and for 1 month after the last immunization	• History of other malignancies over the last 5 years (except basal cell carcinoma of the skin or carcinoma *in situ* of the bladder)<br>• Uncontrolled medical condition considered as high risk for treatment with an investigational drug, including unstable diabetes mellitus, vena-cava-syndrome, known ascites, and/or pleural effusion, symptomatic pleural effusion treated by puncture<br>• Renal insufficiency requiring dialysis<br>• Patients who are planning to father a child/not willing to use a barrier method of contraception

AA, anti-androgen; ALT, alanine aminotransferase; AST, aspartate aminotransferase; ECOG, Eastern Cooperative Oncology Group; GCP, good clinical practice; HBV, hepatitis B virus; HCV, hepatitis C virus; HIV, human immunodeficiency virus; LH-RH, luteinizing hormone-releasing hormone; NYHA, New York Heart Association; PCWG, Prostate Cancer Working Group; PSA, prostate-specific antigen; RECIST, Response Evaluation Criteria in Solid Tumors; ULN, upper limit of normal

Table S3: Selected epitopes of CV9103 antigens

	PSA	PSCA	PSMA	STEAP1
**A1**	VSHSFPHPLY<br>PSLYTKVVHY<br>ALPERPSLY	LQPGTALLCY<br>CVDDSQDYY	HSTNGVTRIY<br>ETYELVEKFY<br>AGDPLTPGY	EHDVWRMEIY<br>WIDIKQFVWY<br>ATSHQQYFY
**A2**	FLTPKKLQCV<br>KLQCVDLHV<br>VISNDVCAQV	ALQPGTALL<br>ALLPALGLL	LLHETDSAV<br>ALFDIESKV<br>VLAGGFFLL	LLLGTIHAL<br>FLYTLLREV<br>LLLGTIHAL
**A3**	GAAPLILSR<br>QVHPQKVTK<br>AVCGGVLVH	AVGLLTVISK<br>RIRAVGLLT<br>ALLMAGLAL	KVFRGNKVK<br>STEWAEENSR<br>RLLQERGVAY	IVQLHNGTK<br>SITLLALVY<br>AIVQLHNGTK
**A24**	CYASGWGSI<br>HYRKWIKDTI	DYYVGKKNI	NYARTEDFF<br>LYSDPADYF	VFLPIVVLI<br>QYFYKIPIL
**B7**	TPKKLQCVDL<br>RPGDDSSHDL<br>GPLVCNGVL	QPAAAILAL	RPRWLCAGAL<br>LPSIPVHPI<br>RPRRTILFA	IVQLHNGTK<br>ILFLPCLRK<br>AILALLAVTS
**B8**	CIRNKSVIL<br>LLKNRFLRP<br>MSLLKNRFL	TARIRAVGL<br>TVISKGCSL	GNKVKNAQL<br>GWRPRRTIL<br>LAGAKGVIL	CLRKKILKI<br>MLTRKQFGL
**B35**	EPALGTTCY<br>HPEDTGQVF	QPGTALLCY<br>QPAAAILAL	DPLGLPDRPF<br>FPGIYDALF	HPLATSHQQY<br>TPPTFMIAVF
**B44**	EEFLTPKKL<br>QEPALGTTCY	GEQCWTARI<br>ARIRAVGLL	NEIFNTSLF<br>KEFGLDSVEL<br>HEIVRSFGTL	DEFDCPSEL<br>SELQHTQEL<br>REFHYIQSKL
**DRB1*01**	TLSVTWIGAAPLILS<br>ASGWGSIEPEEFLTP	AAILALLPALGLLL<br>AVLLALLMAGLALQP	FFLLGFLFGWFIKSS<br>QKLLEKMGGSAPPDS	SFFFAVLHAIYSLSY<br>LLALVYLPGVIAAIV
**DR B1*03**	CGGVLVHPQWVLTAA<br>NKSVILLGRHSLFHP	SLNCVDDSQDYYVGK<br>ILALLPALGLLLWGP	ISIINEDGNEIFNTS<br>GVILYSDPADYFAPG	DKWMLTRKQFGLLSF<br>SYPMRRSYRYKLLNW
**DR B1*04**	HSLFHPEDTGQVFQV<br>QVFQVSHSFPHPLYD	AVLLALLMAGLALQP<br>KAQVSNEDCLQVENC	STEWAEENSRLLQER<br>VKNFTEIASKFSERL	LTFLYTLLREVIHPL<br>PGVIAAIVQLHNGTK
**DR B1*07**	VPVVFLTLSVTWIGA<br>HPQWVLTAAHCIRNK	LNCVDDSQDYYVGKK<br>ILALLPALGLLLWGP	GMVFELANSIVLPFD<br>MKTYSVSFDSLFSAV	IAAIIASLTFLYTLL<br>IHALIFAWNKWIDIK
**DR B1*011**	PSLYTKVVHYRKWIK<br>TGQVFQVSHSFPHPL	VGLLTVISKGCSLNC<br>EQCWTARIRAVGLLT	LYNFTQIPHLAGTEQ<br>CRDYAVVLRKYADKI	KIPILVINKVLPMVS<br>YTLLREVIHPLATSH
**DR B1*015**	LQGITSWGSEPCALP<br>SRIVGGWECEKHSQP<br>KKLQCVDLHVISN	LALLMAGLALQPGTA<br>AHALQPAAAILALLP	SDIVPPFSAFSPQGM<br>HEIVRSFGTLKKEGW	VWRMEIYVSLGIVGL<br>TFMIAVFLPIVVLIF
**Promiscuitive epitopes**	WQVLVASRGRAVCGG<br>SVTWIGAAPLILSRI	TARIRAVGLLTVISK<br>AHALQPAAAILALLP<br>ILALLPALGLLLWGP	VKNFTEIASKFSERL<br>NYTLRVDCTPLMYSL	LSFFFAVLHAIYSLS<br>INKVLPMVSITLLAL

SA, prostate-specific antigen; PSCA, prostate stem cell antigen; PSMA, prostate-specific membrane antigen; STEAP1, 6-transmembrane epithelial antigen of the prostate 1

Table S4: Summary of all immunologically evaluable patients. Response against antigen, type of detected response, time of detected response and fold increase above baseline are summarized.

Pt	Dose	antibody		CD4				CD8			
		±	Week of max	±	Antigen	Week of max	Max fold	±	Antigen	Week of max	Max fold
1	I	neg		neg				pos	PSA	17	4.33
								pos	PSMA	9	3
								pos	STEAP1	17	5
2	I	neg		neg				neg			
3	I	neg		neg				neg			
4	II	neg		neg				neg			
5	II	pos	17	neg				neg			
6	III	neg		neg				pos	PSA	17	8
7	III	neg		pos	PSA	9	2.56	neg			
8	III	pos	9	neg				neg			
9	III	neg		neg				neg			
10	III	pos	17	pos	PSMA	9	2	neg			
11	III	neg		pos	PSMA	17	7	neg			
					STEAP1	17	8.25				
12	III	neg		neg	STEAP1	17	2	neg			
13	III	neg		neg				neg			
14	III	neg		pos	PSA	9	8	neg			
					PSCA	9	2				
					STEAP1	17	3				
15	III	neg		neg				neg			
16	III	neg		pos	PSCA	5	2	neg			
17	III	neg		pos	PSA	17	2	neg			
					PSCA	9	17.6				
					PSMA	17	25				
					STEAP1	17	34.5				
18	III	neg		neg				pos	PSMA	17	3.33
									STEAP1	17	5.6
19	III	neg		neg				neg			
20	III	neg		pos	PSMA	9	2.2	pos	PSA	9	3.5
21	III	neg		neg				pos	PSA	9	2.39
									PSMA	17	3.83
22	III	neg		neg				neg			
23	III	neg		pos	PSMA	17	44	pos	PSMA	17	15.63
					STEAP1	17	21		STEAP1	17	11.13

24	III	neg		neg				neg			
25	III	neg		neg				pos	PSA	9	2.75
									PSCA	5	4
26	III	neg		pos	PSMA	17	3	neg			
27	III	neg		neg				pos	PSA	17	126
									PSMA	9	3
									STEAP1	17	2
28	III	neg		pos	PSA	17	8	pos	PSA	17	8
				pos	PSCA	9	7		PSCA	9	3
				pos	STEAP1	9	4		PSMA	9	4
				neg					STEAP1	9	22.6
29	III	pos	17	neg				pos	PSA	5	3.88
30	III	neg		pos	PSA	9	3.05	pos	PSCA	9	2
				pos	PSMA	9	15.4				
31	III	neg		pos	PSMA	9	8.45	pos	PSA	5	7
32	III	neg		neg				pos	PSCA	17	2
									PSMA	17	2
									STEAP1	17	2.25
33	III	pos		neg				pos	PSCA	9	2.53
									PSMA	9	2.5
34	III	neg		pos	PSA	9	9	neg			
35	III	neg		neg				pos	PSA	9	14.5
36	III	neg		neg				neg			
37	III	neg		neg				pos	STEAP1	9	4
38	III	neg		neg				pos	PSMA	17	9

CD, cluster of differentiation; max, maximum; mRNA, messenger ribonucleic acid; PSA, prostate-specific antigen; PSCA, prostate stem cell antigen; PSMA, prostate-specific membrane antigen; STEAP1, 6-transmembrane epithelial antigen of the prostate 1

Table S5: Number of subjects receiving 1, 2, 3, 4, or 5 vaccinations per cohort

Dose level/number of subjects	I (N=3)	II (N=3)	III (N=38)	All (N=44)
1 vaccination				
2 vaccinations		1	1	2
3 vaccinations			2	2
4 vaccinations	2		7	9
5 vaccinations	1	2	28	31

Table S6: Antigen-specific responses for patients responding to only one antigen.

	PSA	PSCA	PSMA	STEAP
Patient 1	+			
Patient 2	+			
Patient 3	+			
Patient 4				+
Patient 5		+		
Patient 6			+	
Patient 7	+			
Patient 8	+			
Patient 9	+			
Patient 10				+
Patient 11			+	

Table S7: Antigen-specific responses for patients responding to two or more antigens.

	PSA	PSCA	PSMA	STEAP
Patient 1	+		+	
Patient 2			+	+
Patient 3	+	+		+
Patient 4	+	+	+	+
Patient 5			+	+
Patient 6	+		+	
Patient 7	+		+	
Patient 8			+	+
Patient 9	+	+		
Patient 10	+		+	+
Patient 11	+	+	+	+
Patient 12	+	+	+	
Patient 13	+		+	
Patient 14		+	+	+
Patient 15	+	+	+	
