# Supplementary material for: Self-adjuvanted mRNA vaccination in advanced prostate cancer patients: a first-in-man phase I/IIa study
Source: J Immunother Cancer. 2015 Jun 16;3:26. doi: 10.1186/s40425-015-0068-y (PMC4468959; doi:10.1186/s40425-015-0068-y)

Figure S1: Immune responses by age category. Immune responses were independent of age. Patients were grouped into patients below age 65 and at or above age 65. Responses in each subgroup are displayed. No significant difference in between these age groups can be detected.

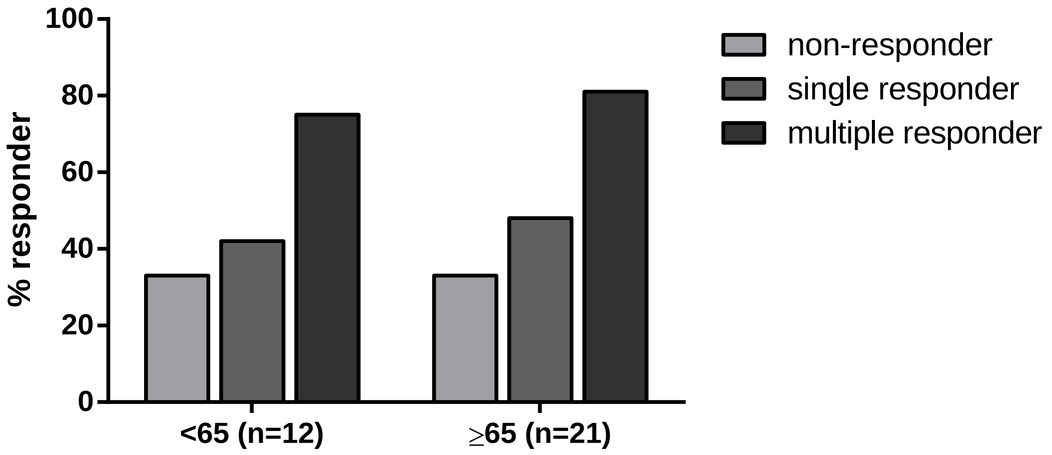

Figure S2: Increase in CD19+ B cells. Frequency of CD19+ B cells at baseline and during vaccination. A non-significant change in frequencies of B cells during the course of vaccination could be detected over time, with an at least two-fold increase at≥1 timepoint of the B cell frequency in 45% of patients.

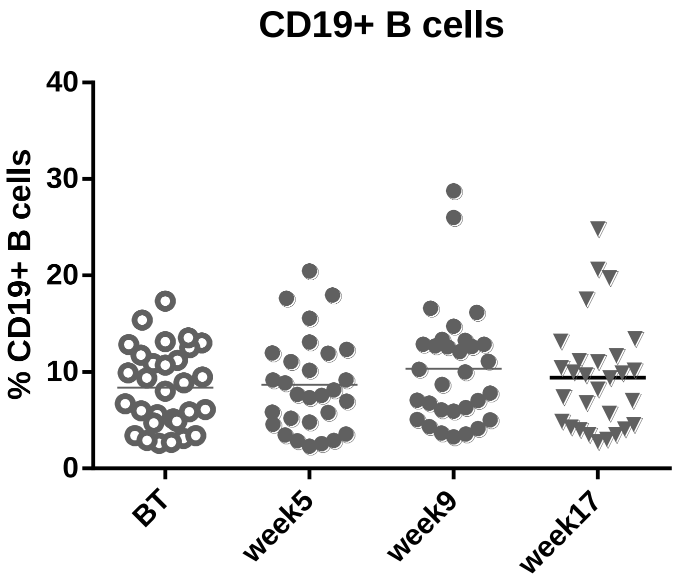

Figure S3: Analysis of regulatory T cells. Frequency of regulatory T cells at baseline and during vaccination. No significant change in frequencies of regulatory T cells during the course of vaccination could be detected, and no difference in the frequency of regulatory T cells between immune responders and non-responders was observed.

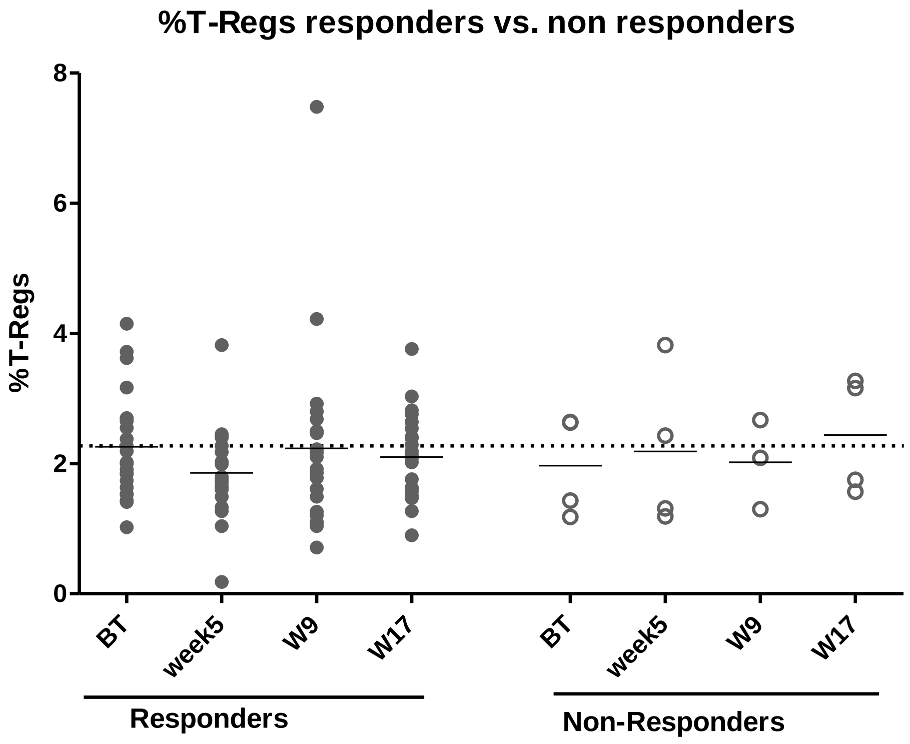

Figure S4: Example assay data

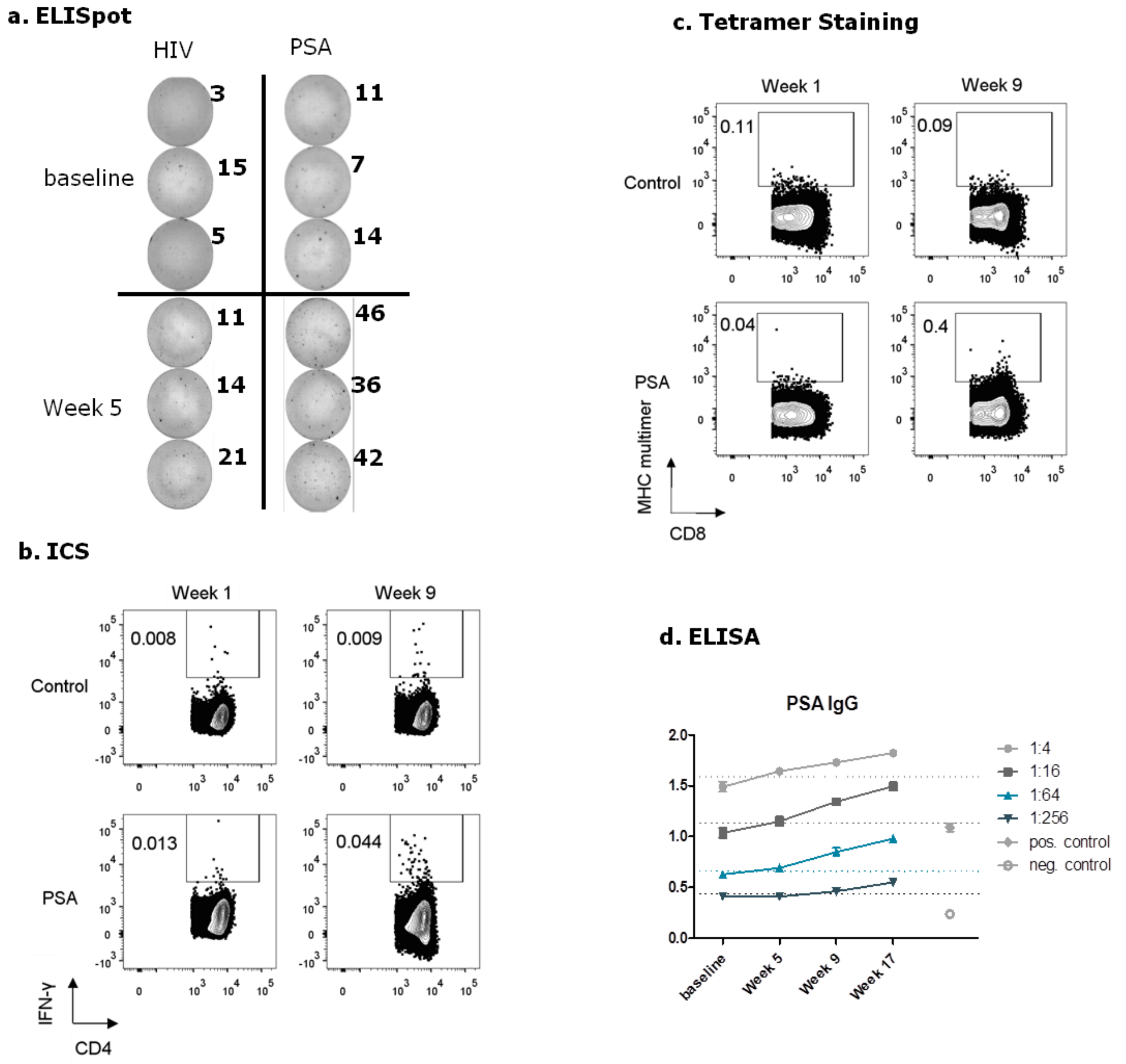

Supplement: Additional file 2: Figure S1. — Immune responses by age category (<65 or ≥65 years old) showing that immune responses were independent of age. Figure S2. CD19+ B cell levels showing anincrease over time. Figure S3. Analysis of regulatory T cells. No change in frequencies of regulatory T cells was seen during the course of vaccination and no difference between responders and non-responders was seen. Figure S4. Example assay data. [file 40425_2015_68_MOESM2_ESM.pdf]
