# Supplementary material for: Self-adjuvanted mRNA vaccination in advanced prostate cancer patients: a first-in-man phase I/IIa study
Source: J Immunother Cancer. 2015 Jun 16;3:26. doi: 10.1186/s40425-015-0068-y (PMC4468959; doi:10.1186/s40425-015-0068-y)
Supplement: Additional file 3: — Case report of a 59 year old patient who developed a confirmed PSA response after initial increase in PSA. [file 40425_2015_68_MOESM3_ESM.pdf]

Case report

A 59 year old patient developed a confirmed PSA response after initial increase in PSA (a). The patient had detectable antibodies against PSA at baseline which did not increase during vaccination (b), in the *ex vivo* IFN-gamma ELISpot assay the frequency of antigen specific T cells against several antigens were increased after baseline but did not fulfill the response criteria (c). In *ex vivo* IFN-gamma ICS assay, increased frequencies or CD4 and CD8 T cells against several antigens were seen that were below the response threshold; response criteria were fulfilled for CD4 PSA week 9 (e.) but not for the other antigens and timepoints (d. and e.).

a.

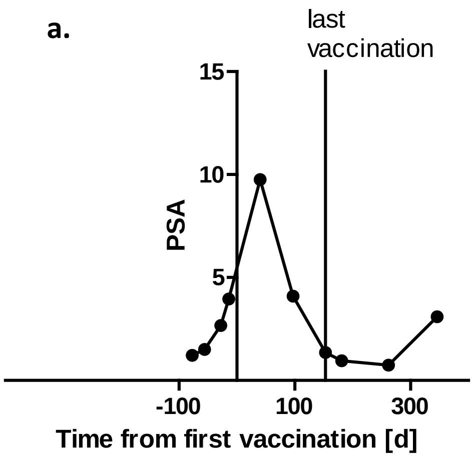

b.

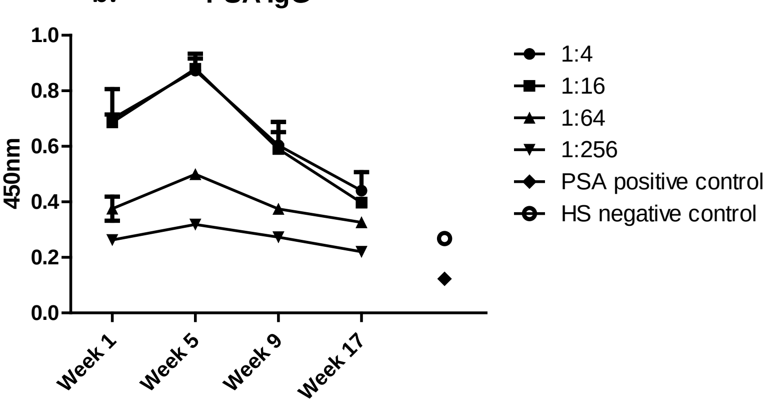

c.

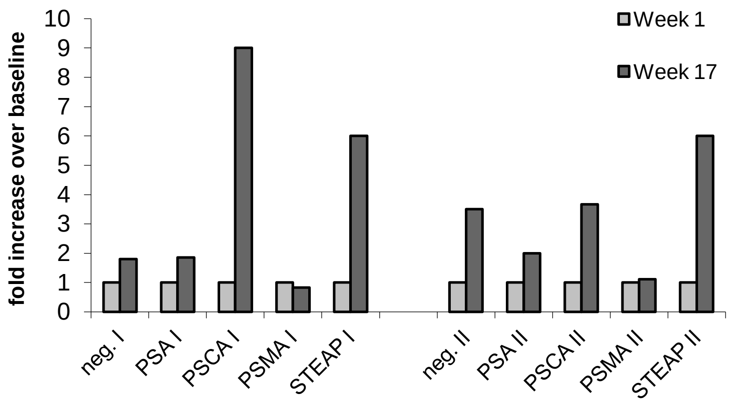

d.

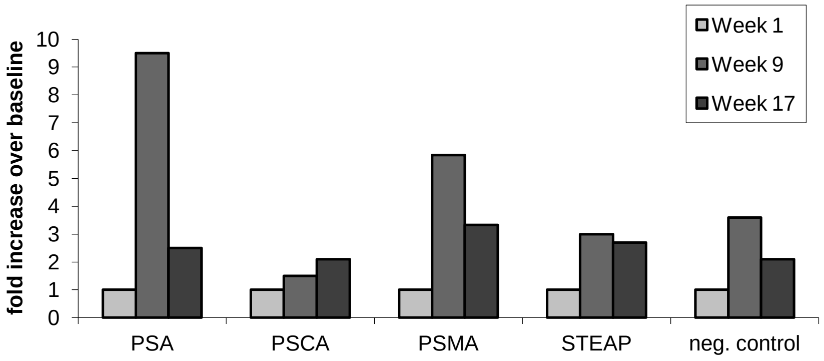

Week 1
Week 9
Week 17
fold increase over baseline
0
1
2
3
4
5
6
7
8
9
10
PSA
PSCA
PSMA
STEAP
neg. control

e.

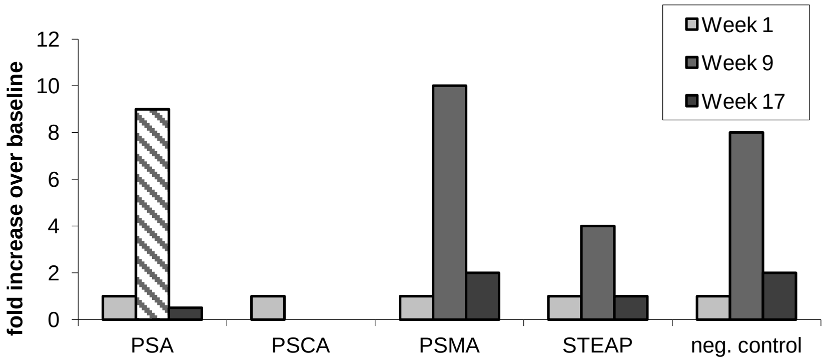

Week 1
Week 9
Week 17
fold increase over baseline
0
2
4
6
8
10
12
PSA
PSCA
PSMA
STEAP
neg. control
