# Supplementary material for: Self-adjuvanted mRNA vaccination in advanced prostate cancer patients: a first-in-man phase I/IIa study
Source: J Immunother Cancer. 2015 Jun 16;3:26. doi: 10.1186/s40425-015-0068-y (PMC4468959; doi:10.1186/s40425-015-0068-y)
Supplement: Additional file 4: Table S8. — List of Ethical Bodies. [file 40425_2015_68_MOESM4_ESM.pdf]

Table S8: List of Ethical Bodies

Site	Ethics Committee
**Germany**	
Charité Universitätsmedizin Berlin<br>Campus Benjamin Franklin<br>Urologische Klinik und Hochschulambulanz	**Ethik-Kommission des Landes Berlin<br>Landesamt für Gesundheit und Soziales (LAGeSo)**
UKSH Campus Lübeck<br>Klinik und Poliklinik für Urologie	**Ethikkommission der Medizinischen Fakultät der Universität zu Lübeck**
Universitätsklinikum Tübingen<br>Klinik für Urologie	**Ethikkommission an der Medizinischen Fakultät und am Universitätsklinikum Tübingen**
Klinikum rechts der Isar der TU München<br>Urologische Klinik und Poliklinik	**Ethik-Kommission der Fakultät für Medizin der TU München**
Klinikum der Johann-Wolfgang-Goethe Universität Frankfurt<br>Klinik für Urologie und Kinderurologie	**Ethikkommission des Fachbereichs Medizin der Johann-Wolfgang-Goethe Universität**
Universitätsmedizin Mannheim<br>Urologische Klinik	**Medizinische Ethik-Kommission II der Medizinischen Fakultät Mannheim**
Johannes-Gutenberg-Universität Mainz<br>Urologische Klinik und Poliklinik	**Ethikkommission der Landesärztekammer**
Universitätsklinikum Essen<br>Klinik und Poliklinik für Urologie, Uroonkologie und Kinderurologie	**Ethik-Kommission der Medizinischen Fakultät der Universität Duisburg-Essen**
Universitätsklinikum Aachen<br>Urologische Klinik	**Ethik-Kommission der Medizinischen Fakultät der RWTH Aachen**
Universitätsklinikum Carl Gustav Carus der Technischen Universität Dresden	**Ethik-Kommission der Medizinischen Fakultät Carl-Gustav-Carus der TU Dresden**
Universitätsklinikum Freiburg<br>Abteilung Urologie	**Ethik-Kommission der Albert-Ludwigs-Universität Freiburg**
**Italy**	
Unità di Immuno-Bioterapia dei Melanomi e dei Tumori Solidi - Fondazione Scientifica Istituto San Raffaele	**COMITATO ETICO DELL´IRCCS FONDAZIONE S. RAFFAELE DEL MONTE TABOR DI MILANO**
